# Experimental and modeling studies on enhancing the thermodynamic hydrate inhibition performance of monoethylene glycol via synergistic green material

**DOI:** 10.1038/s41598-021-82056-z

**Published:** 2021-01-27

**Authors:** Arul Bharathi, Omar Nashed, Bhajan Lal, Khor Siak Foo

**Affiliations:** 1 Department of Chemical Engineering, Universiti Teknologi PETRONAS, 32610 Bandar Seri Iskandar, Perak Malaysia; 2 CO2 Research Centre (CO2RES), Universiti Teknologi PETRONAS, 32610 Bandar Seri Iskandar, Perak Malaysia; 3 PTTEP, Level 26-30, Tower 2, Petronas Twin Towers, Kuala Lumpur City Centre, 50088 Kuala Lumpur, Malaysia

**Keywords:** Energy science and technology, Engineering

## Abstract

This paper presents an experimental and modeling studies on the thermodynamic inhibition effects of the mixture of monoethlyene glycol (MEG) and glycine (Gly) on the carbon dioxide hydrate phase boundary condition. The monoethlyene glycol and glycine (1:1) mixture inhibition effects were investigated at concentrations of 5, 10, and 15 wt.% and pressure ranges from 2.0–4.0 MPa. The effects of the proposed mixture on the carbon dioxide hydrate phase boundary were evaluated by measuring the dissociation temperature of carbon dioxide hydrate using a T-cycle method. The synergistic effect was evaluated based on comparison with pure MEG and Gly data. The results show that 15 wt.% of MEG and Gly mixture displays the highest inhibition effect compared to the 5 and 10 wt.% mixtures, respectively. However, the synergistic effect is higher at 10 wt.%. Dickens' model was also adopted to predict the phase equilibrium data of CO_2_ hydrates in the presence of the mixture. The modified model successfully predicted the data within a maximum error of ± 0.52 K.

## Introduction

Gas hydrates are ice-like inclusion compounds formed when water and gas come into contact at high pressure and low-temperature conditions^1^. The guest gas molecules such as carbon dioxide are then trapped in the cages of the hydrogen-bonded water molecules^2,3^. There are three main types of gas hydrates crystal structures, which are; (1) structure I (sI), (2) structure II (sII), and (3) structure H, (sH). The presence of water vapor in natural gas is the root cause of hydrate formation; a phenomenon, which significantly affects the transportation of natural gas and poses a major challenge to the oil and gas industry^4–7^. Gas hydrate formation in natural gas production may block the production pipelines which potentially affects the production facilities, and eventually hinder natural gas production^8,9^. The progressive field development of oil and gas explorations into deep waters (> 500 m); exposes production pipelines to hostile operating environments which are prone to gas hydrate formation^10^. Especially when handling natural gas compositions rich in water-soluble gases such as CO_2_. The presence of CO_2_ increases the hydrate formation risk compared to methane or other gases because it forms hydrate a lower pressure conditions^11^.

To prevent hydrate formation, the system's thermodynamic operating conditions (temperature and pressure), are controlled so that the system does not reach the hydrate stability zone^1,12^. Currently, methods such as depressurization, injection of chemical additives, dehydration, and heating could be used to mitigate gas hydrate formation^13^. However, chemical injection is widely used due to its economic advantage and technological viability^14^. The chemical injection method involves the injection of additives to prevent the formation of gas hydrates during the transmission of hydrocarbons in pipelines^15^.

There are three types of chemical inhibitors used to control gas hydrate formation. They are; thermodynamic inhibitors (THIs) and, kinetic inhibitors (KHIs) and, anti-agglomerates (AAs)^16^. This research paper primarily focuses on THIs, including ways to significantly enhance their performance to benefit the oil and gas industry. This is because THIs are the most-primary type of inhibitors used in natural gas production facilities. THIs mainly inhibits hydrate formation by shifting the hydrate phase equilibrium boundary condition to high pressure and low temperature conditions^17^. An effective THI shifts the hydrate region by forming hydrogen bonds with water molecules to avoid the formation of hydrate cages. Although THIs are good additives for mitigating hydrates, they are used in large amounts (10–50 wt.%) which prohibits their application. The high concentrations utilized are costly and might be environmentally challenging^18,19^. The most effective and applicable THIs are methanol (MeOH) and monoethlyene glycol^20^. Several attempts have thus been made to find alternative inhibitors that are efficient, environmentally friendly, and cost-effective. Ionic liquids have been proposed as dual function gas hydrates inhibitors as they can inhibit the formation of hydrates both kinetically and thermodynamically^21^. Ionic liquids (ILs) possess unique characteristics such as low vapor pressure, tunable structures, and high thermal and chemical stability^20^. However, ILs have low thermodynamic inhibition performance compared to commercial inhibitors such as methanol^22^. Amino acids as green gas hydrate inhibitors have attracted more attention since Sa et al. reported their inhibition impact on CO_2_ hydrates^23^. Amino acids are capable of forming hydrogen bonds with water molecules due to the presence of both amine and carboxyl groups in their structure^16,22,24^. Like ionic liquids, amino acids have not been proven to compete and replace commercial THIs.

In view of this, researches have been conducted to examine the performance of combining different thermodynamic inhibitors^24^. The combination of ionic liquids with ethylene glycol as well as with other ionic liquids provides a strong synergistic thermodynamic inhibition effect at high pressure^23,25^. Recently, amino acid combinations with MEG have been tested as gas hydrates inhibitors. Mixing glycine with MEG boosts the thermodynamic inhibition impact compared to their pure performances for methane hydrates^26^. Another study revealed that L-tyrosine enhances the kinetic inhibition of MEG + PVP mixture^27^. The use of glycine as an inhibitor is advantageous due to its unique characteristics, such as; excellent biodegradability, hydrophilicity, cost-effectiveness, corrosion inhibition characteristics, and lastly suitable hydrates inhibition qualities^23,28,29^. However, their limited solubility at high concentration and thermodynamic inhibition performance encourage the researcher to utilize them as co-inhibitors with conventional inhibitors. To the best of our knowledge, very limited scientific reports have studied the synergistic effect of glycine on conventional THIs. Moreover, CO_2_ tends to form hydrates faster than hydrocarbons due to its high solubility in water; thus, a severe operational problem is often encountered during the production of CO_2_ rich natural gas. Given that the phase equilibrium is a function of the type of gas, there is an imperative need to have such data, especially there is no software that can predict MEG + Gly phase equilibrium. The chosen chemical mixture with different concentrations has not been investigated for CO_2_ hydrate inhibition in the existing literature. Therefore, quantitative analysis is required to evaluate the synergistic effect of glycine on MEG on CO_2_ hydrate and as well predict the hydrate phase behavior of CO_2_ hydrate in MEG and glycine (1:1) mixtures.

In this work, a mixture of MEG and glycine was chosen and experimented as an efficient thermodynamic hydrate inhibitor. The CO_2_ hydrate dissociation temperature was measured using an isochoric step heating method in a sapphire hydrate cell reactor. The synergistic effect is studied and compared with individual inhibitors. Quantitative analysis was carried out by calculating the molar hydrate dissociation enthalpies using the Clausius–Clapeyron equation. The enthalpy of hydrate dissociation (H_d_) could help to identify the hydrates structure^30^. Finally, Dickens’ model was adopted in this study to predict the equilibrium temperature of CO_2_ hydrates in the presence of the mixture.

## Results and discussion

### Phase equilibrium of carbon dioxide hydrate

**Figure 1:**
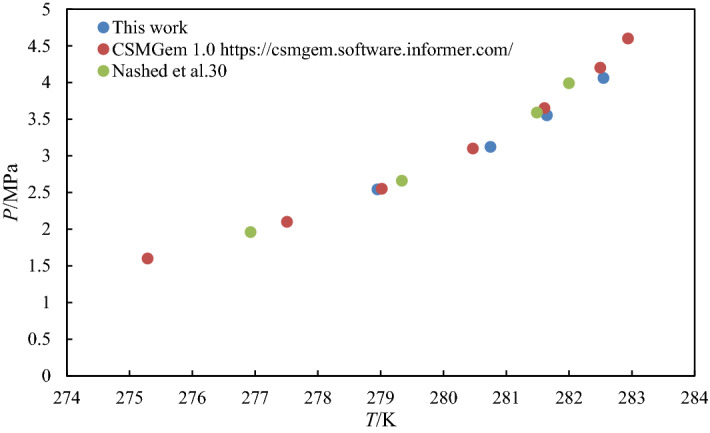
Comparison of CO_2_ hydrates equilibrium conditions of pure water with CSMGem 1.0 software and literature^31^.

**Figure 2:**
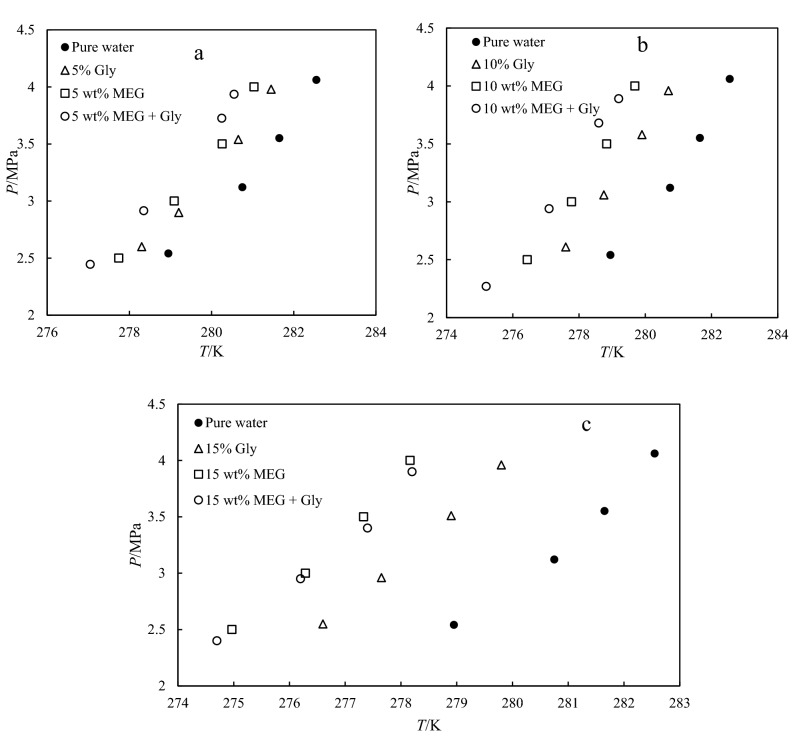
HLVE curve of CO_2_ hydrate for pure water and (**a**) 5wt.% of THIs (**b**) 10 wt.% of THIs, and (**c**) 15 wt.% of THIs.

The hydrate dissociation temperature is identified during the slow heating step. The CO_2_ hydrates phase equilibrium conditions are in the pressure ranges of 2.0–4.0 MPa and temperature ranges of 274.70–282.55 K. To verify the reliability of the methodology, the dissociation hydrates temperature of the blank sample was measured and compared with literature and predicted data by CSMGem software. The results show a good agreement with the published data as plotted in Fig. 1, thus, validating the accuracy of the experimental methodology adopted in this study. Figure 2 presents the measured CO_2_ hydrates phase equilibrium conditions in the presence of 5, 10. and 15 wt.% mixtures (1:1) of MEG and glycine with uncertainty of ± 0.01. Figure 2 also compares the effect of pure MEG, glycine, and their mixtures on the CO_2_ hydrates phase behavior. It is observed that all tested systems were able to shift the equilibrium curve toward a lower temperature zone, thus, confirming their inhibition effect. The presence of carboxyl and amine groups in the structure of glycine could act as hydrogen bond donors and acceptors. Therefore, glycine forms hydrogen bonds with water molecules preventing them to link together to form the hydrate crystals. For MEG, it is well known that the two hydroxyl groups present in its structure form strong hydrogen bonds with water molecules, thus accounting for its hydrate inhibition impact. It is worth to mention that the smaller molecular size of MEG exhibit high charge density compared to amino acids that have alkyl chains. According to Fig. 2, the 1:1 mixture of MEG + Gly shifts the carbon dioxide hydrate dissociation curve towards the lower temperature region. The thermodynamic hydrate inhibition effect of the mixture is higher at 5 and 10 wt.% compared to those of the individual components present in the mixture.

Based on the results shown in Fig. 2, we can deduce that the conventional inhibitor has higher inhibition strength than glycine; however, the mixture of MEG + Gly, gives a more satisfying result, which could be useful to the industry. The findings also confirm that amino acids can synergistically improve the performance of THIs, such as MEG.

**Figure 3:**
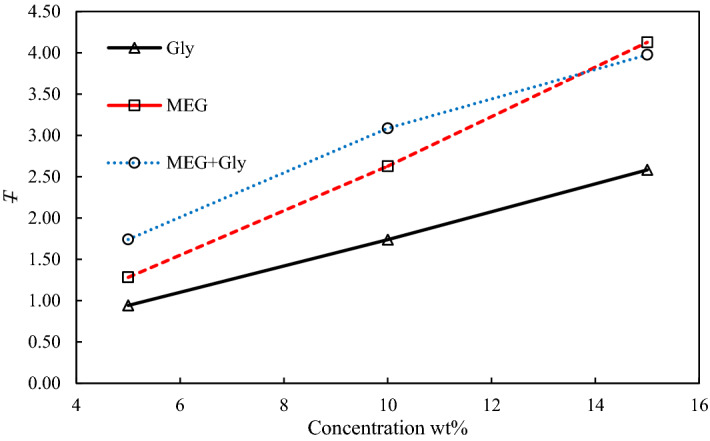
Comparison of average reduced temperature in the presence of THI solutions.

The average reduced CO_2_ hydrate equilibrium temperature was calculated using Eq. (1), to estimate the hydrate inhibition impact of the THIs quantitatively^32–34^. The estimated average reduced temperature results are shown in Fig. 3.

1 $$ T = \frac{1}{n}\mathop \sum \limits_{i = 1}^{n} \Delta T $$

where *n* is the number of data points, and *ΔT* is the difference between the equilibrium point in the presence of pure water and MEG or glycine or their mixtures at a specified pressure. A comparison was also made between the mixture and its pure component, as depicted in Fig. 3. The MEG data were obtained from CSMGem software while that of glycine was obtained from our previous work^23^. Based on Fig. 3, it is observed that the inhibition impact is more pronounced at 15 wt.%, which confirmed the direct proportional of the thermodynamic inhibition to the concentration. It can be observed that the effectiveness of 1:1 mixture at 5 and 10 wt.% is higher than the pure components. However, at 15 wt.%, MEG outperforms the mixture. The crossover occurs near 13.7 wt.% where the impact of MEG would be equal to 1:1 mixture. The inhibition impact could be due to the combination of strong hydrogen bonds offered by MEG and the functional groups of glycine (NH_3_^+^ and COO^-^), which interacts electrostatically with the other water molecules.

It is worth mentioning that having glycine in the system causes an additional advantage to the system due to its kinetic inhibition effect. The chemical interactions between MEG-glycine is complicated and different compared to MEG-water and Gly-water interaction. Glycine has carboxyl and amine groups which act as hydrogen bond donors and acceptors. In addition, it has the ability to transfer one proton within its molecule to form zwitterion in aqueous solution, enhancing its charge density, hence, increase the density of hydrogen bond donors and acceptors per unit volume. The zwitterionic character of the amino acid gives it a high attraction to the dipolar water and MEG molecules. Subsequently, the presence of mixed inhibitors in the solution disrupts hydrogen bonding, which reduces the favourability of the oriented hydrate crystal structures. On the other hand, MEG tends to form intermolecular hydrogen bonds within one molecule due to the attraction between its two hydroxyl groups due to the molecular geometry. Having an extra-strong charge around the molecules could reduce the effect of this phenomenon and enhance the attachment of hydroxyl groups to water or glycine. As a result, the average intermolecular interaction between both inhibitors with water molecules is more potent than a single inhibitor solution. This subsequently causes more hydrate inhibition compared to pure MEG or glycine.

Contrary, when the concentration of the glycine is increased in the15 wt.% mixture, the synergistic effect is reduced. This is because the amino acid is less polarity compared to MEG and water, hence, the solubility behavior of the amino acids is altered by providing an environment of continuously decreasing polarity due to the addition of a semi-polar liquid to the polar aqueous solvent. As the concentration of glycine is increased, the solvent medium would continually decrease the polarity and glycine could be in equilibrium with its uncharged molecular form due to its decreased aqueous content or polarity in the system. This phenomenon could affect the synergic effect of increasing glycine concentration on the system’s water activity favorable to cause additional hydrate inhibition as observed in the experimental results in this work. The absence of synergic effect between glycine and MEG at high glycine concentration could also be due to the fact that glycine tends to form its own hydrates as the glycine molecule could be surrounded by seven water molecules. This is attributed to favorable stable hydrate formed due to the absence of strong glycine-glycine and water-water interaction^35^, thus causing a negligible synergic inhibition impact.

The mixture of MEG and glycine showed better inhibition performance compared to each inhibitor; thus, the existence of the synergistic effect is investigated. This can be done by comparing the average reduced temperature of the mixture at a given concentration within the arithmetic mean *T*_*a*_ of the average reduced temperature for the individual components, as shown in Eq. (2):

2 $$ T_{a} = \frac{1}{2}\left( {T_{ M} + T_{ G} } \right) $$

where *T*_*M*_ and *T*_*G*_ are the average reduced temperature of MEG and glycine at the same inhibitor concentration, respectively. Hence, a similar total concentration is considered in this calculation by assuming that two non-interacting inhibitors have an equal contribution as the mixture is 1:1. It is also worth to mention that Richard and Adidharma reported that summing the impact of individual inhibitors of concentration w/2 to evaluate the synergistic effect is inaccurate as doubling the concentration of a single inhibitor does not lead to double average reduced temperature^25^.

**Figure 4:**
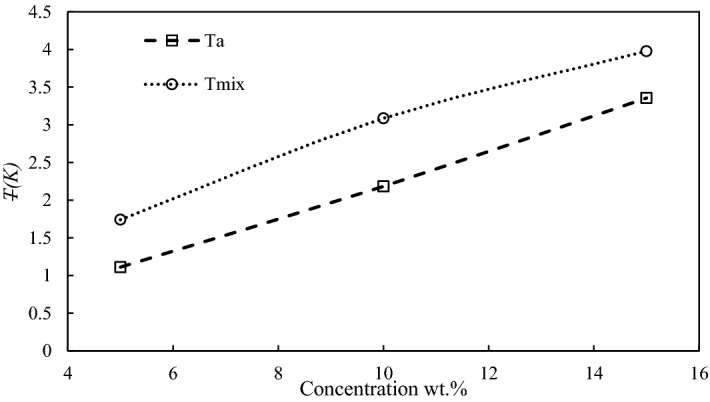
The effect of concentration on the average reduced temperature of 1:1 MEG + Gly mixture and the arithmetic mean of the reduced temperature of the individual components.

Figure 4 demonstrates the effect of concentration on average reduced temperature of MEG + Gly (*T*_mix_) and the arithmetic mean of reduced temperature (*T*_*a*_) of individual inhibitors. The synergistic effect is obvious at all studied concentrations as *T*_*mix*_ is higher than *T*_*a*_. However, it is more pronounced at 10 wt.%. Eventually, it is recommended to run experiments at high concentrations to identify the effective range for a synergistic effect.

### Molar carbon dioxide hydrate dissociation enthalpy

**Figure 5:**
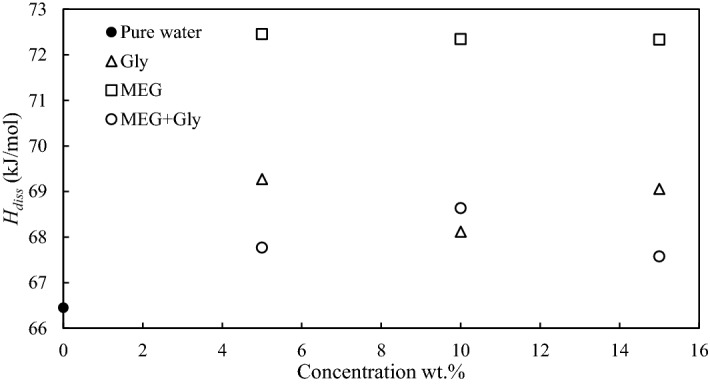
Comparison of enthalpy of CO_2_ hydrate dissociation in the presence of 10wt.% of THIs and pure water.

The results are further studied using the Clausius–Clapeyron equation [Eq. (3)] to determine the thermal properties of carbon dioxide hydrate by calculating the molar hydrate dissociation enthalpy of CO_2_ in the presence of the proposed THI mixture^36^.

3 $$ \frac{d\ln P}{{d({1 \mathord{\left/ {\vphantom {1 T}} \right. \kern-\nulldelimiterspace} T})}} = - \frac{\Delta H}{{zR}} $$

where *P* is the system pressure, *T* is temperature, *ΔH* is the enthalpy of hydrate dissociation, z is the compressibility factor which is calculated based on the Peng-Robinson equation of state, and *R* is the universal gas constant. The ratio *dlnP*/*d*(1*/T*) is the slope of the line produced by the plotting of *lnP* against 1/*T*. The hydrate dissociation is an endothermic process as the energy required to break the hydrate crystals. Therefore, it is a function of crystal hydrogen bonding, hydrate stability, and cavity occupation. The enthalpy of hydrate dissociation is shown in Table 1 and Fig. 5, the results show that the hydrate formed in the presence of MEG and glycine are within structure I enthalpy for CO_2_ hydrates. The values in the presence of the mixture do not change significantly compared to water or with respect to the concentration. It can be concluded that the inhibitor does not affect the type of hydrate structure, but only encourages the reduction of water activity to prevent hydrate formation. However, it is common to observe a slight increase of enthalpy in the presence of inhibitors, which might be due to the increase of Gibbs free energy, which requires a higher pressure or lower temperature to form a hydrate^37^.

**Table 1:** Molar dissociation enthalpies of carbon dioxide hydrate in the absence and presence of different chemical additives.

Water	MEG + Gly		
	5 wt.%	10 wt.%	15 wt.%
**Average ΔH/kJ mol**^**-1**^			
66.451	67.77	68.64	67.58

Thus, by analyzing the enthalpy of carbon dioxide hydrate dissociation in the absence and presence of different chemical additives, it is found that maximum energy is used to destruct the hydrogen bonds between the water molecules in the hydrate structure. A thermodynamic hydrate inhibitor prevents the formation of hydrates by destroying water activity through hydrogen bonding^38^. The inhibition mechanism of combined inhibitors reduces molecule activity, thus increase the competition for water molecules. This situation occurs due to the hydrophobic effect by water molecules to prohibit nonpolar molecules. The strong electrostatic interactions formed between our combined inhibitor reduces the formation of hydrates. From Fig. 5, we can observe that conventional inhibitor, pure MEG needs more energy to break the hydrogen bonds compared to the suggested inhibitor thus our proposed THI is highly recommended to consider in the gas industry. However, an increase in Gibbs free energy results in a minor rise of the hydrate dissociation enthalpy. The most accurate way to determine the heat of dissociation (ΔH) is to measure it calorimetrically^39^.

### Thermodynamic modeling

The proposed model is applied to predict the influence of the studied THIs on the carbon dioxide hydrate equilibrium phase condition. The model predictions average absolute errors (AAE) are calculated using Eq. (4) to evaluate the fitting of the model.

4 $$ AAE = \frac{1}{m}\sum\limits_{i = 1}^{m} {\left| {T_{Exp.} - T_{Cal.} } \right|}_{i} $$

**Figure 6:**
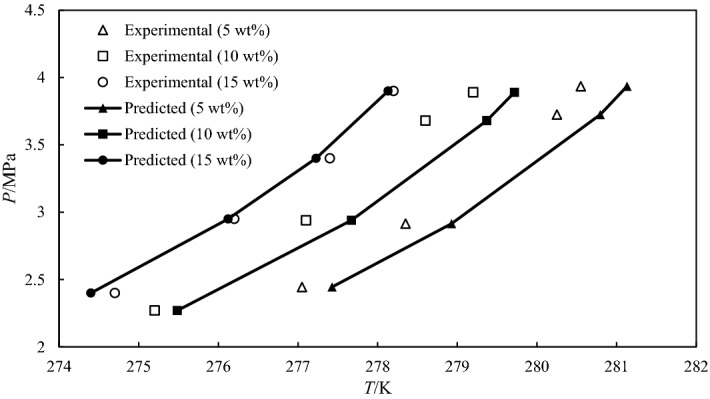
Predicted and experimental CO_2_ hydrate dissociation temperature in the presence of 1:1 mixture of MEG + Gly.

According to Fig. 6, the model predictions gives good agreement with the experimental data with an AAE of 0.15–0.52 K (Table 2). This further validates the accuracy of the model. The application of the model for modeling the hydrate phase behavior in THIs for any gas hydrate former is recommended.

**Table 2:** Predicted carbon dioxide hydrate equilibrium points in the presence of different THIs.

Sample	No. data point	Temperature range (K)	AAE (K)
MEG + Gly (5 wt.%)	4	277.42 – 281.13	0.52
MEG + Gly (10 wt.%)	4	275.48 – 279.71	0.49
MEG + Gly (15 wt.%)	4	274.39–278.13	0.15

The solubility of CO_2_ in water produces more ions which can affect the equilibrium temperature. However, this model does not take into account the amount of gas dissolved in the liquid, which could result in higher error. It also concluded that the freezing depression temperature plays a major role in this model. Hence, special attention should be paid to adopt the van't Hoff factor to get accurate predictions.

## Experimental

### Chemicals

The selected chemicals; monoethlyene glycol and glycine, are purchased from Merck, and they are used without further purification. Deionized water was dispensed from the Ultra-Pure Water System, Model: Lab Tower EDI 15 and used for the preparation of the sample solutions. An equal proportion 1:1 of MEG and Gly based on mass fraction are added to prepare the mixture solutions at 5,10, and 15 wt.%. Samples are weighted using an analytical balance with the accuracy of ± 0.001 g. The above-stated chemicals are experimented in the presence of carbon dioxide gas with purity of 99.95% supplied by Air Product SDN BHD.

### Apparatus and procedure

**Figure 7:**
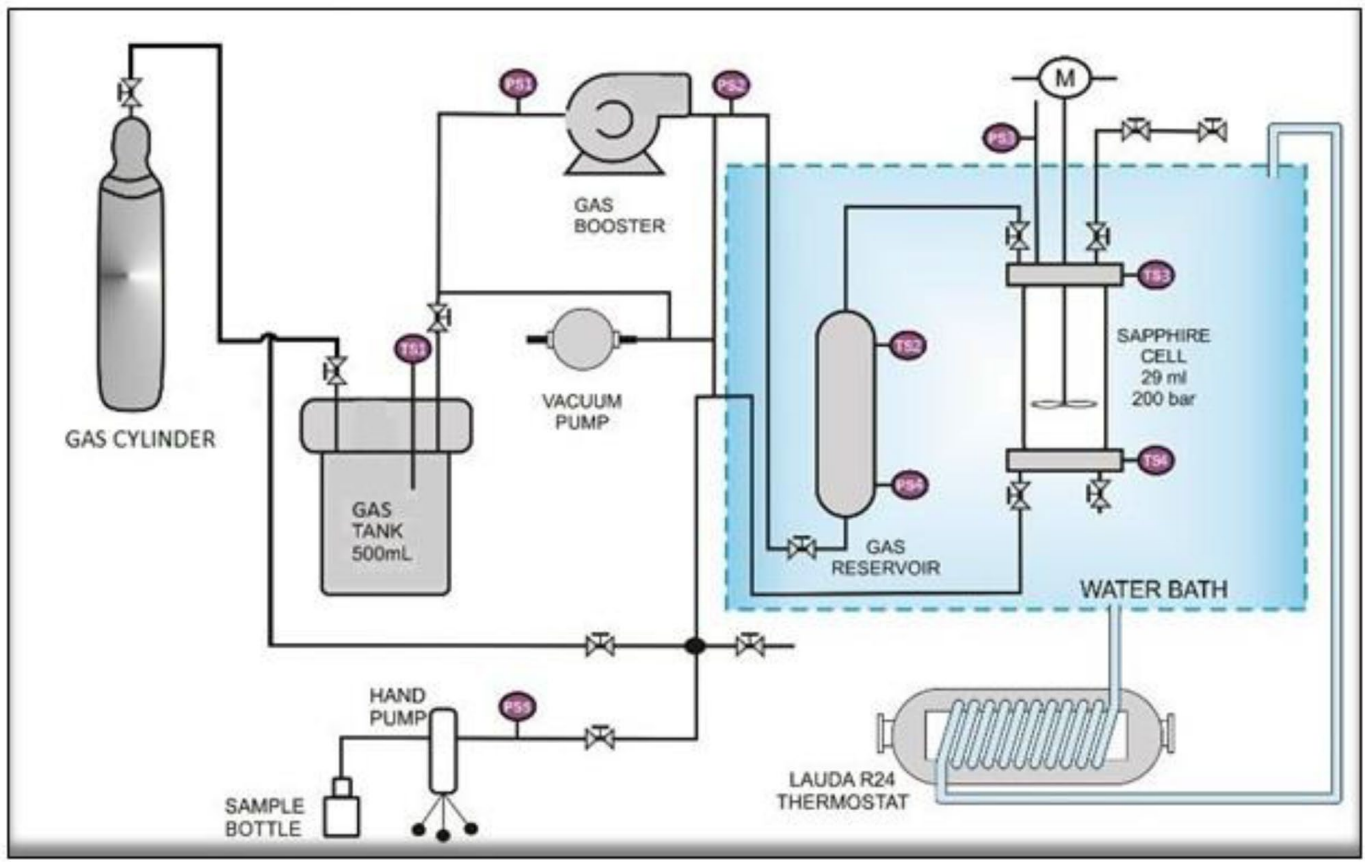
Schematic diagram of the experimental apparatus in this work^41^.

**Figure 8:**
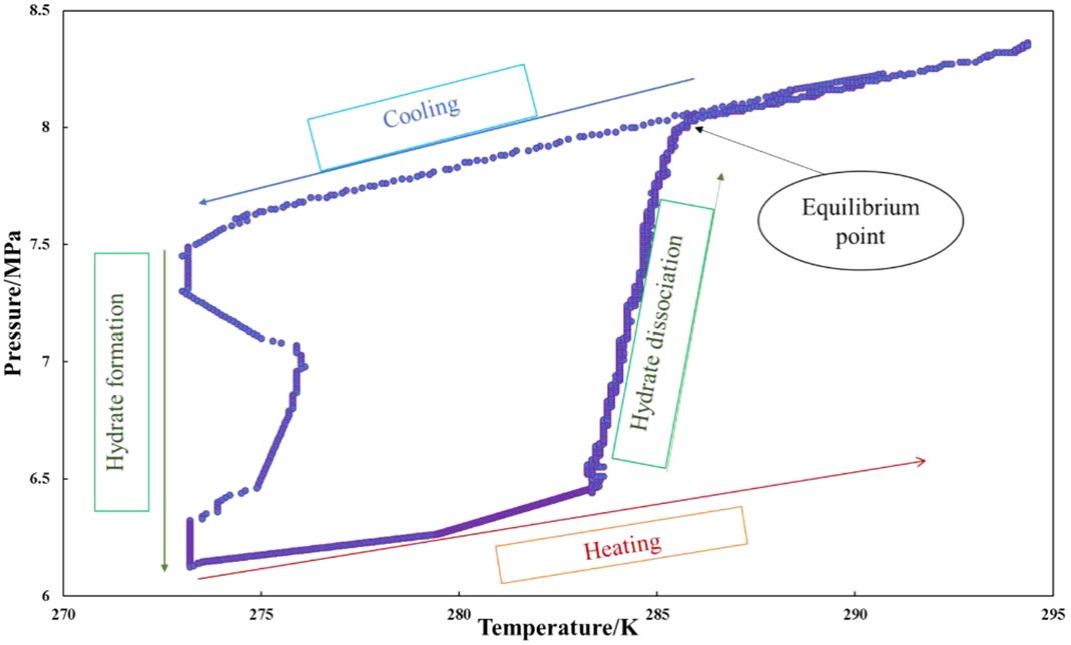
T-Cycle method to measure the equilibrium point.

A traditional T-cycle method with isochoric step heating technique was implied in this research work^40^. The equipment used for this study is a sapphire hydrate cell reactor. Figure 7 shows the schematic diagram of the apparatus. The apparatus consists of 29 ml transparent sapphire cells, in which experiments can be monitored by physical observation. The setup is designed to function up to 20 MPa, and temperature ranges of 275.2 to 280.55 K. The sapphire cells are placed in the reactor, and the temperature of the cells is controlled by its respective thermostats. To ensure adequate stirring, a magnetic torque stirrer is placed in the cell. The whole apparatus is connected to a data acquisition system that continuously records pressure and temperature in the cell every 10 s with an accuracy of ± 0.01 MPa and ± 0.01 K, respectively. The prepared chemical mixture will be injected into the cell, and then it is pressurized at the desired pressure. T-cycle methods illustrated in Fig. 8 is employed to get hydrates dissociation temperature, which represents the hydrate equilibrium point. In this method, the temperature was reduced rapidly up to 273.15 K and kept constant until complete hydrates formation. A sudden pressure drop indicates the formation of hydrates. Then, a stepwise heating method was applied with an average heating rate of 0.25 K/hr. The slow heating rate is crucial for detecting the accurate hydrate dissociation temperature. The interception of the P–T curve during heating with its cooling curve is determined at hydrate equilibrium data. To establish the equilibrium curve, the experiments were repeated at not less than four pressure points.

### Theory

Only a few thermodynamic models can be applied to predict the phase equilibria of gas hydrates in the presence of chemical inhibitors. However, a model proposed by Dickens and Quinby-Hunt, that involves the effect of chemical additives on the activity of water gives a precise result. Hence, the model is adopted to be used to estimate the equilibrium points of CO_2_ hydrates in the presence of thermodynamic hydrate inhibitors. This model is applicable for CO_2_ hydrate since the authors applied it on methane hydrates where methane and carbon dioxide hydrates are classified as structure I hydrate^36^. The model is the adaptation of the Pieroen model where the author formulated a relationship between the enthalpy of formation, water activity, and suppression temperature. Below is the formula used to calculate the activity of THIs solutions [Eq. (5)]^36^:

5 $$ Ina_{w} = \frac{{\Delta {\rm H}_{FUS\left( i \right)} }}{R}\left[ {\frac{1}{{T_{f\left( i \right)} }} - \frac{1}{{T_{f} }}} \right] $$

where *a*_*w*_ denotes water activity, *ΔH*_*FUS(i)*_ is the heat of fusion of ice (6.008 kJ/mol), *T*_*f(i)*_ and *T*_*f*_ are the freezing point temperatures of pure water (273.15 K) and THI solution, respectively. *T*_*f*_ is calculated, as suggested by Dickens and Quinby-Hunt [Eq. (6)]^36^, using a cryoscopic constant for water as 1.853 K·kg/mol.

6 $$ \Delta T_{f} = K_{F} \cdot m.i $$

where *ΔT*_*F*_ is the freezing point depression, *K*_*F*_ is the cryoscopic constant, which considered 1.86 K kg/mol for water, m is the molality (mol/kg), and I is the van't Hoff factor.

Where the van't Hoff factor represents the ratio between the actual number of moles of particles produced in solution per mole of solute, it matches the actual concentration of all species produced by the solute after dissolution. For non-electrolytes material where the solute does not dissociate, thus, van't Hoff factor is essentially 1.0. For weak electrolytes that cannot perform 100% dissociation such as amino acid, the van't Hoff factor can be ranged between 1 and 2. To predict the freezing point of glycine solutions, van 't Hoff factor was considered as 1 according to the literature^16,41,42^. When glycine mixes with MEG, the non-ideality of the system increases due to the interaction between glycine and MEG. Consequently, the freezing point depression changed. However, it could not be the sum of freezing point depression of individual solutes. Therefore, in this work, the van't Hoff factor of mixed inhibitor solutions was taken as 1.5 to calculate the freezing point depression. This value was chosen because glycine could produce NH_2_-CH_2_-COO^-^ and H_3_O^+^ when it dissolved in the water-MEG mixture along with the uncharged form NH_2_-CH_2_-COOH. As it was explained, MEG and glycine have a synergistic impact on each other, and MEG could enhance the production of charged glycine rather than the uncharged form. Therefore, the number of van't Hoff factor assumed to be 1.5 to represent the amount of glycine being partially ionized into two ions and the amount of glycine remain unchanged, which agrees with the statement that weak electrolyte could have 1–2 van't Hoff factor*.*

The effect of THIs on the carbon dioxide hydrate dissociation temperature, can be represented as below [Eq. (7)]:

7 $$ Ina_{w} = \frac{{\Delta {\rm H}_{d} }}{nR}\left[ {\frac{1}{{T_{w} }} - \frac{1}{{T_{THI} }}} \right] $$

where *H*_*d*_ represents the dissociation enthalpy of carbon dioxide hydrates, *R* is the universal gas constant, and *T*_*w*_ and *T*_*THI*_ are the hydrate formation temperatures in pure water and aqueous THIs solution respectively. CSMGem software was used to calculate the pure water, glycine, and MEG—carbon dioxide hydrate dissociation temperature.

The combination of Eqs. (5–7) describes the temperature offset of carbon dioxide hydrate phase condition and the temperature of the ice-water equilibrium condition in any THIs solution at constant pressure and below formula [Eq. (8)] can be used to calculate *T*_*THI*_:

8 $$ \left[ {\frac{1}{{T_{w} }} - \frac{1}{{T_{THI} }}} \right] = \frac{{n\Delta {\rm H}_{FUS\left( i \right)} }}{{\Delta {\rm H}_{d} }}\left[ {\frac{1}{{T_{f\left( i \right)} }} - \frac{1}{{T_{f} }}} \right] $$

Table 3 below shows the calculated *T*_*f*_ values of aqueous THI solution systems.

**Table 3:** The freezing point temperatures (K) of aqueous THI solutions in different concentrations.

Thermodynamic hydrate inhibitor	Pure glycine			Pure MEG			MEG + Gly		
Concentration (wt.%)	5	10	15	5	10	15	5	10	15
*T*_*f*_ (K)	271.77	270.40	268.78	271.57	269.82	267.86	270.99	268.59	265.90

## Conclusion

Carbon dioxide hydrate phase conditions in the presence of monoethlyene glycol and glycine have been investigated in an isochoric sapphire hydrate cell reactor. Carbon dioxide hydrate inhibition effect was observed in the presence of the combined inhibitor with different concentrations. The proposed mixture at 15 wt.% has a very high inhibition strength due to their strong hydrogen bonding interaction with water molecules. The influence of our combined inhibitor prevents the thermodynamic formation of hydrates more than individual inhibitors. The synergistic effect is highest at 10 wt.% of 1:1 mixture solution. The dissociation enthalpy results indicate that inhibitors do not change the hydrate structure. Moreover, the mixture could be dissociated with less energy compared to MEG. Finally, the Dickens’ model predicted the equilibrium temperature with maximum AAE = 0.52 K, suggesting that the model can be adopted for predicting the hydrate phase behavior for mixed inhibition systems as well as for pure inhibitors upon proper modification.
